# Supplementary material for: Terminal complement complexes with or without C9 potentiate antimicrobial activity against Neisseria gonorrhoeae
Source: mBio. 2025 Mar 31;16(5):e00141-25. doi: 10.1128/mbio.00141-25 (PMC12077172; doi:10.1128/mbio.00141-25)
Supplement: Supplemental Figures — Fig. S1-S3. [file mbio.00141-25-s0001.pdf]

Supplemental Figure 1

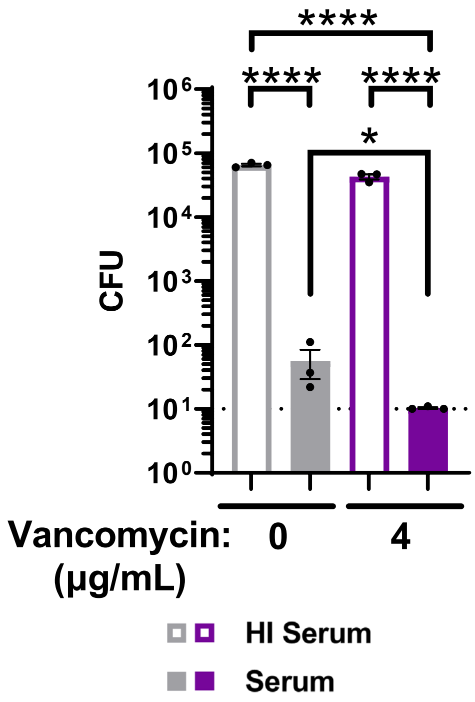

Supplemental Figure 1. Vancomycin activity is potentiated in the MS11 strain of Gc. MS11 Gc pre-incubated with anti-gonococcal IgM followed by incubation with 2% IgG/M-depleted human serum with or without heat-inactivation (HI), and subsequent incubation with 4µg/mL vancomycin or vehicle alone for CFU enumeration. Error bars are SEM. Significance was determined by 1-way ANOVA with Tukey's multiple comparisons on $\log_{10}$-transformed data. * = $p<0.05$, **** = $p<0.0001$. Dotted line represents minimum reportable CFUs.

Supplemental Figure 2

Gc Strain

FA19

Minimum Inhibitory Concentration (MIC)		
Nisin		µg/mL
Serum	0%	50-100
Concentration:	2.5%	0.39-0.78

MS11

Minimum Inhibitory Concentration (MIC)		
Ceftriaxone		ng/mL
Serum	0%	8.0-16.0
Concentration:	2.5%	0.0625

FA1090

Minimum Inhibitory Concentration (MIC)		
Ceftriaxone		ng/mL
Serum	0%	2.0-8.0
Concentration:	2.5%	0.0625-0.25

FA19

Minimum Inhibitory Concentration (MIC)		
Ceftriaxone		ng/mL
Serum	0%	0.25-0.5
Concentration:	2.5%	0.03125-0.0625

Supplemental Figure 2. Minimum inhibitory concentrations of nisin and ceftriaxone are decreased by serum across multiple Gc strains. Additional Gc strains were assayed via 16hr MIC broth microdilution assays over a range of nisin or ceftriaxone concentrations in media alone or supplemented with 2.5% IgG/M-depleted human serum.

Supplemental Figure 3

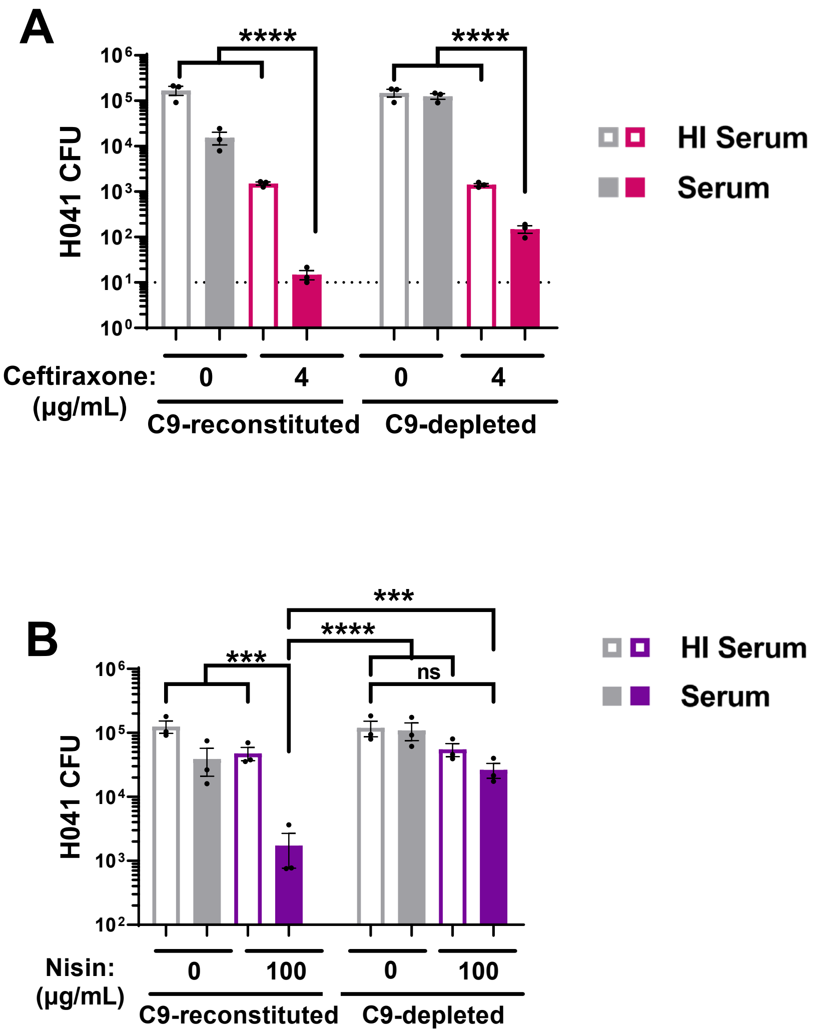

Supplemental Figure 3. Complement C5b-C8 complexes and full C5b-C9 MAC differentially potentiate ceftriaxone and nisin against Gc. H041 Gc was pre-incubated with anti-gonococcal IgM, followed by incubation with 1% C9-depleted or C9-reconstituted human serum with or without heat-inactivation (HI). Gc was then incubated with ceftriaxone (A) or nisin (B) and then plated for CFU enumeration. Error bars are SEM. Significance was determined by 1-way ANOVA with Tukey's multiple comparisons on log_{10}-transformed data. *** = p<0.001, **** = p<0.0001. ns = not significant. Dotted line represents minimum reportable CFUs.
